# Stability Qualification of Resins/Metallic Oxide Composites for Surface Oxidative Protection

**DOI:** 10.3390/polym16030333

**Published:** 2024-01-25

**Authors:** Traian Zaharescu, Radu Mirea, Tunde Borbath, Istvan Borbath

**Affiliations:** 1 INCDIE–ICPE CA, Radiochemical Center, 313 Splaiul Unirii, Ro 030138 Bucharest, Romania; 2 ROSEAL SA, 5A Nicolae Bălcescu, Harghita District, Ro 535600 Odorheiu Secuiesc, Romania; borbath.tunde@gmail.com (T.B.); borbathistvan@roseal.eu (I.B.); 3 Romanian Research and Development Institute for Gas Turbines-COMOTI, 220D Iuliu Maniu Bd., Ro 061125 Bucharest, Romania; radu.mirea@comoti.ro

**Keywords:** resin composite, metallic oxide filler, degradation, chemiluminescence

## Abstract

The accelerated degradation of alkyd resins via γ-irradiation is investigated using non-isothermal chemiluminescence. The stability qualification is possible through the comparison of emission intensities on a temperature range starting from 100 °C up to 250 °C under accelerated degradation caused by radiolysis scission. The measurements achieved in the samples of cured state resin modified by various inorganic oxides reveal the influence of metallic traces on the aging amplitude, when the thermal resistance increases as the irradiation dose is augmented. Even though the unirradiated samples present a prominent chemiluminescence intensity peak at 80 °C, the γ-processed specimens show less intense spectra under the pristine materials and the oxidation starts smoothly after 75 °C. The values of activation energies required for oxidative degradation of the sample subjected to 100 kGy are significantly higher in the composite states than in the neat resin. The degradation mechanism of polymerized resins is discussed taking into account the effects of fillers on the stability of studied epoxy resin at various temperatures when the degradation and crosslinking are in competition for the decay of free radical.

## 1. Introduction

The addition of metallic oxides in the structure of polymer composites determines the increased material stability in most of the cases [1,2,3,4]. The general overview on the issued papers presenting the improved properties of polymer composite is rather focused on the functional properties, because the majority of assessments principally depict the contributions of fillers to efficient applications [5,6,7,8]. The modifications in the basic functional features related to the interaction between the two compounding phases, namely organic matrix (polymer) and inorganic structure (filler), are caused by the interphase conditioning at the boundary surface. Accordingly, the modification in energetic differences between the conduction and the valence bands influences the stability of the polymer material [9]. Some papers have identified this kind of intimate correlations [10,11] as the jointing degradation fragments of macromolecules that exist in the neighborhood of specific structured lattices as often happens during the radiation processing of polymers [12]. The composites containing metallic oxides present good resistance against burning due to their intumescent capacity for the limitation of fire propagation [13]. The main contributions of oxide fillers included in polymer materials are differentiated by the dissimilarity of electronic densities between the metallic atoms, as well as the high electronegativity of oxygen that makes the formation of gaps with an electronic deficit possible [2].

In the cases of structured composites, the control of interphase cohabitation plays a determinant role on the initiation and propagation of oxidation [14]. The compatibility between polymer chains with the surrounding filler particles is sustained by various interactions: hydrogen and van der Waals bonds, Coulomb interactions, adsorption and superficial scavenging, and suitable diffusion into the free volume of polymer structures [15]. Accordingly, the size of attraction degree, the local concentration of degradation intermediates, the pretreatment of particles or the conditions of material processing lead to the necessity of an attentive analysis of the peculiar characteristics determining the amplitudes of changes [16]. The interaction between the filler particles and polymer macromolecules is illustrated by various aspects of structural modifications like tribological properties [17], energy conversion [18], electrical conductivity [19], and many other performances.

Among the different ways through which polymer materials are subjected to advanced oxidation (heating, photodegradation or the exposure to high-energy irradiation), the radiation processing may provide deeper and more relevant transformations [20]. The attraction of free radicals formed during radiolysis by the active centers that exist on the particle’s surface may delay oxidation and, consequently, extend the material durability [21]. However, in these additives/fillers, there are some inorganic structures that behave as pro-oxidants [22].

The stabilization assisted by oxide fillers is always connected to the activity of defects that are present in the material’s lattice [23]. The presence of this kind of materials with imperfect lattices in the formulation of polymer composites brings a certain convenient evolution of oxidation. When the reacting intermediates are involved in the product aging or in the association with surface defects, an improvement in the oxidation state is obtained. The metallic oxides are always involved in the polymer conditioning through their labile electrons, which allow for the occupation of a conduction band by foreign electrons belonging to the free radicals. The unpaired electrons are scavenged in the deep gaps and, consequently, the oxidation is hindered. In contrast, the propagation of degradation during the oxidation of polymers is possible due to the role of diffused oxygen [24].

**Figure 1:**
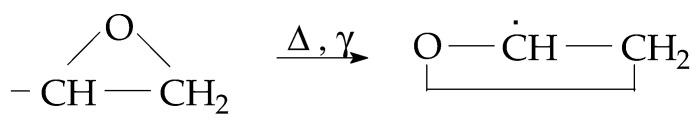
The main structure that influences the stability of epoxy resins.

The radiolysis of resins represents an accelerated degradation when the stressing agents act through a high local energetic transfer that deposits an excess of organic molecules [25]. The fragmentation of the molecular chains takes place during the exposure to high-energy irradiation, when several low-energy bonds are broken. Accordingly, the new free radicals become available for oxidation, and the degradation products based on oxygenated functions are progressively accumulated. The increase in their amounts demonstrates the availability of the aging material for the propagation of oxidation [26]. There are various methods for the delay of degradation: the addition of specific stabilizers like antioxidants [27,28], inorganic nanoparticles [29,30], and crosslinking [31]. The stability of epoxy units, three-atom rings (Figure 1), influences the start and the progress of oxidative degradation, which are dependent on the sample’s composition [32].

After the examination of thermal degradation on the stability of some epoxy resins, the values of activation energies were reported [33]. The mean energy required for the oxidation of this kind of compounds is 144 kJ mol^−1^ when the inspected materials were totally fragmented through pyrolysis. The structure is converted into another independent unit, which is further decomposed and oxidized. During γ-radiolysis, the molecular chains are also split, where highly substituted carbon atoms are placed as it occurs in the macromolecules of highly branched polyethylene (LDPE) [34] or bisphenol A [35]. A detailed discussion of the degradation of DGEBA (bisphenol A diglycidyl ether) was previously presented in [36], as an example for our material, when the accelerated degradation under the action of γ-ray is applied.

The presence of oxides in the compositions of polymer-based materials is a determining factor for modifications in long-term stability. While some inorganic fillers like oxides or clays extend the durability ranges [2,37,38], other compounds behave as pro-oxidants [39]. However, the nanocomposites with specific fillers have peculiar commitments, which are related to the extension of application ranges under different energetic stressing conditions (nuclear engineering, transports, aircraft, pipe sealing).

The present paper presents a detailed analysis of the influence of the metallic surface composition on the stability of applied protective layers in relation to the integrity of organic sheet. The added oxides simulate the presence of alloying elements which interact directly with the epoxy resin coating. The analysis of the chemiluminescence results may indicate the contribution of the metallic structure to the durability of the conserved products.

## 2. Materials and Methods

The pristine epoxy resin was delivered by RESOLTECH (Château d’Arc, France) as product 1050, a liquid product with two components. The main characteristics are adapted from the curing base resin to a laminated product by means of a specific hardener (1054 s) by applying a blending ratio of 100:35 *w*/*w*. The former characteristics of epoxy resin are as follows: density 1.19 g cm^−3^, viscosity @ 23 °C: 1300 mPa s^−1^, and gelation time @ 23 °C: 14 h. The cured resin has the following Shore D hardness values: 14 days @ 23 °C: 86 (at T_o_ = 56 °C), and 16 h @ 60 °C: 89 (at T_o_ = 77 °C). These dried materials were obtained after the mixing of liquid resin, the hardener and the appropriate ratio for the concentration of 1 wt% in respect to the dried sample weight. The following oxides were embedded in the resin matrices: CeO_2_, Cr_2_O_3_, Gd_2_O_3_, MgO, MnO_2_, Nd_2_O_3_, and NiO. All of them were manufactured by Merck (Darmstadt, Germania) as pro-analysis-grade products. They were subjected to dry milling for 8 h, when the particle size became less than 100 nm.

After the gelation of each investigated composition, small chops were subjected to advanced degradation via γ-irradiation in a radiation processing machine (Ob Servo Sanguis, Budapest, Hungary) through panoramic exposure. This radiation processing is considered accelerated degradation, which causes a random scission of molecules and the generation of free radicals able to be oxidized if the diffusion of oxygen is stimulated by the increasing temperature [40]. These values are the low and medium technological exposures that are often selected for the processing of polymers [41]. For epoxy resins, these doses are suitable because they generate a convenient concentration of reactive free radicals, and, after irradiation, their decay takes place according to the selected route [42]. The total received doses are 25, 50 and 100 kGy at a dose rate of 0.5 kGy h^−1^. The unirradiated materials were considered references because they are the primary state of material before their service duty of covering metallic surfaces. The oxidation states of the degraded samples were characterized by non-isothermal chemiluminescence under four different regimes of thermal heating rates: 5, 10, 15 and 20 °C min^−1^. The CL device was produced by the Institute of Polymer, Slovak Academy of Sciences, Bratislava, Slovakia). These measurements were accomplished immediately after the end of the irradiation periods with the aim of avoiding any uncontrolled modification due to the reactions of short-life radicals. Chemiluminescence is a versatile method for the detection of major modifications in the distribution of hydroperoxides that appear after the reactions of free radicals with diffused oxygen during the propagation stage of oxidative degradation, for example, in epoxy resins [43]. The proportionality between the amounts of peroxyl radicals present in the sample at a certain moment of degradation allows for the interpretation of CL spectra that describe the evolution of oxidative degradation [44].

The activation energies were calculated using the Kissinger method [45] by having the input data the onset oxidation temperatures that characterize the theoretical start of oxidation at the four heating rates.

## 3. Results

During the non-isothermal Cl measurements, the increasing temperature produces the corresponding higher values of intensities, which indicate advancing oxidation [46]. The resulting spectra recorded on each solid patterns show the accumulation manner of the intermediates, which generate the final products of oxidation. According to the oxidation scheme based on the radical mechanism [47], the polymer changes its oxidation state as the decay of the intermediates converts them into non-emitting structures. Because the CL intensity value is proportional with the concentration of hydroperoxides, the precursors of all oxygenated degradation products, the shape of the CL spectra illustrates the damage depth.

**Figure 2:**
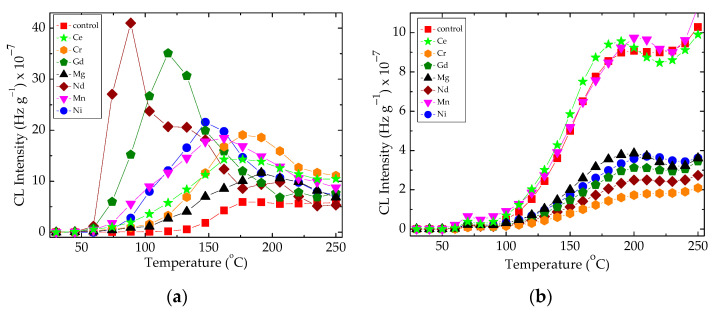
The non-isothermal CL spectra recorded on the epoxy resin modified with metallic oxides. Experimental conditions: (**a**) irradiation dose: 0 kGy, heating rate: 15 °C min^−1^; (**b**) irradiation dose: 100 kGy, heating rate: 5 °C min^−1^.

The influence of the filler on the progress of oxidation in the epoxy resin probes is illustrated in Figure 2. The added oxide particles contribute to the minimization of conversion rates through the decay of hydroperoxides.

**Figure 3:**
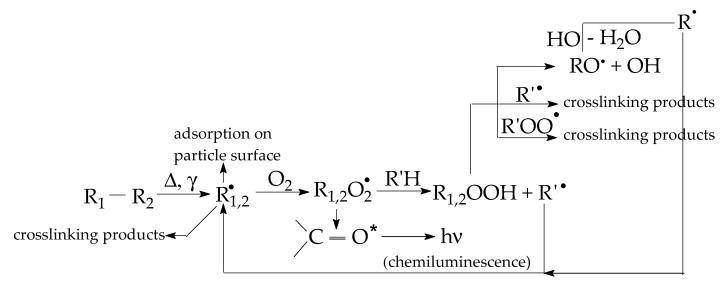
The oxidation pathways from initial structure to degradation products.

The sample degradation trends to separate the family of non-isothermal CL spectra by the maximum emission intensities (Figure 2a) or by the oxidation rates on the medium (100–200 °C) temperature range (Figure 2b). These differences appear in the non-irradiated samples (Figure 2a) when the basic organic material is not yet fragmented, or in the samples after their 100 kGy γ-exposure, when the present filler may be involved into the propagation of oxidation due to the interaction between the degradation intermediates. The oxidative degradation of polymer phase is initiated by the scission of molecules (Figure 3), when weaker bonds are broken, thus providing the early precursors. During radiation exposure, the local concentration of peroxyl radicals along the radiation tracks becomes high, feeding the degradation chains. Based on the radiolysis mechanism initially proposed by Bolland and Gee [48], the proportion of radicals that supports oxidation or recombination depends on the activity of the filler particles.

**Figure 4:**
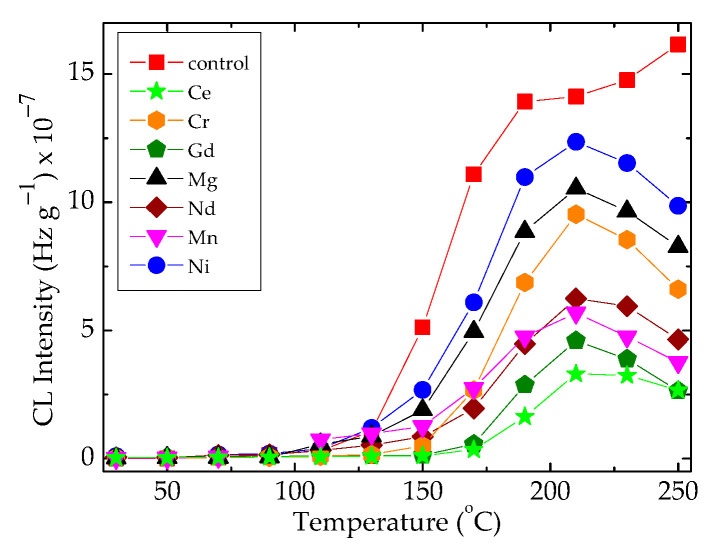
The non-isothermal CL spectra recorded on the EPDM/oxide composites subjected to 50 kGy γ-irradiation by measuring at 20 °C min^−1^.

The superficial interaction between free radicals and the scavenging points belonging to the oxide configuration leads the radicals onto the recombination reaction, when the heating rate reaches 20 °C min^−1^ (Figure 4). The importance of these results consists of the selection of appropriate technological parameters when these materials are processed for the manufacturing of sealing equipment. In an opposite situation, the composite systems consisting of epoxy resin modified with organic materials like bamboo fibers would be placed [49], which require the presence of an efficient antioxidant for a sharp diminution in the oxidation degree. The presence of hydrophobic silica nanoparticles may protect metallic surface through the minimization of the spreading of free radicals inside the polymer substrate [50].

The γ-irradiation of the sheets of epoxy resin/metallic oxides at 100 kGy may be considered a suitable treatment for the hardening of insulation layers and a practical way to be followed when corrosion protection must resist for a long time. The high energy exposure of epoxy resin makes modifications in the structural morphology possible via the jointing of molecular chains on the radical positions [36].

The initiation of oxidative degradation starts in the polymer phase as the result of a second-order reaction between free radicals and diffused molecular oxygen during the propagation stage. In the non-isothermal CL measurements, the increase in sample temperature accelerates the photon emission, which explains the faster accumulation in the oxidation providers: hydroperoxides [51]. As the result of the interaction between oxide particles and radical intermediates, the fresh-born free radicals are scavenged by the particle surface. They become attached to the oxide area and their next stage depends on the jointing strength. If they remain fixed on the filler particles, the CL emission maintains a low intensity. If caught radicals are detached from the filler phase, the oxidation progresses as illustrated by the increase in the CL intensity values. All the prominent maxima demonstrate the highest amounts of intermediates that are oxidized. The descendant part of the CL spectra indicates the decay of a certain amount of hydroperoxides whose gradients are proportional with the diminution in the concentration of radicals.

**Figure 5:**
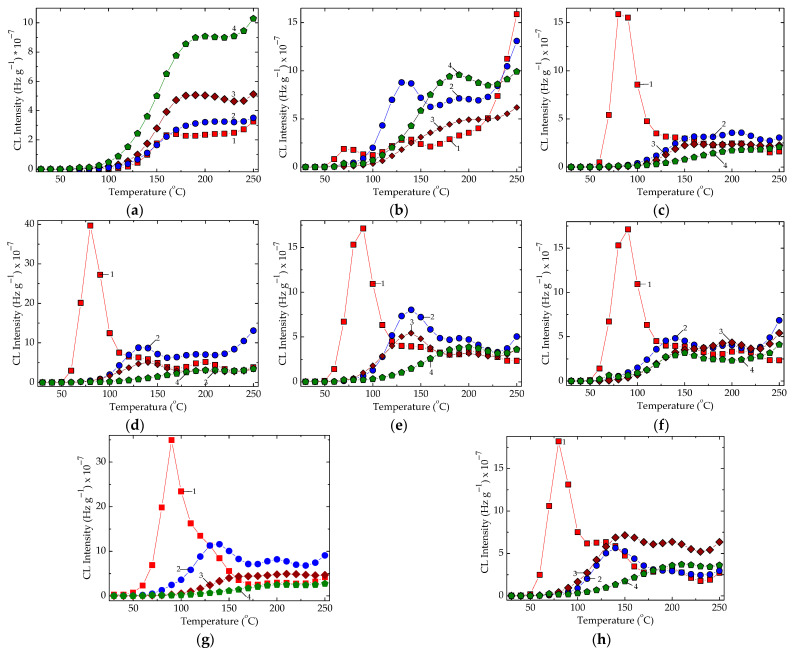
The non-isothermal CL spectra recorded on various epoxy-resin-based compositions containing metallic oxides. Heating rate: 10 °C min^−1^. Irradiation doses: (1) 0 kGy, (2) 25 kGy; (3) 50 kGy, (4) 100 kGy. (**a**) Control, (**b**) CeO_2_, (**c**) Cr_2_O_3_, (**d**) Gd_2_O_3_, (**e**) MgO, (**f**) MnO_2_, (**g**) Nd_2_O_3_, (**h**) NiO.

In Figure 4, the differences between the maximum intensities recorded at 210 °C indicate the corresponding amounts of hydroperoxides, which are formed in the presence of various metallic oxides. The noticeable discrepancies allow for the evaluation of the contribution degrees through which the studied fillers influence the degradation rates of composite. The radiation treatment destroys the hydroperoxides formed in many investigated systems (Figure 5c–h). Then, this operation may be considered an appropriate procedure for diminution in corrosion damage on the covered surfaces.

The presence of oxide particles changes the shapes of the CL spectra and the relative positions in respect to the control samples (Figure 5). While the irradiation doses are higher, meaning that the epoxy resin is more profoundly damaged, the most studied systems present reverse sequences, except the composites containing cerium dioxide (Figure 5b). This demonstrates that the particle surfaces become active through the activation of the interphase interaction, which hinders the progress of oxidation by the scavenging free radicals, the generators of the final degradation products.

The γ-irradiation of the sheets of epoxy resin/metallic oxides at 100 kGy may be considered a suitable treatment for the hardening of insulation layers and a practical way to be followed when corrosion protection must resist for a long time. The high energy exposure of epoxy resin makes modifications in the structural morphology possible via the jointing of molecular chains on the radical positions [51].

## 4. Discussion

The degradation process that occurs in base epoxy resin composites is the result of the competition effects, where scission and crosslinking play peculiar roles. In the presence of an oxide filler like alumina, the chain fragments surround the nanoparticles and oxidation is delayed [52]. The estimation of the lifetime of epoxy resin composites may be carried out starting from the values of activation energies calculated for the oxidative degradation of systems [53], which is the main kinetic parameter characterizing the strength of the material.

The oxidation of epoxy resin composites progresses either through molecular fragmentation and the formation of an epoxy structure (Figure 1), or through the scission of one of the four bonds involving quaternary carbon atoms due to the low bond energy [54]. The thermal performances of these materials are based on their resistance, whose activation energies are around 70–75 kJ mol^−1^ [55]. The involvement of oxide filler in the stabilization of polymers against oxidation is characterized by the surface interaction, where the generated free fragments are scavenged by the superficial traps [2,56]. After the initiation of oxidative degradation, the propagation stage may be influenced by the blending filler, which withdraws reactive radicals from the oxidation chains [40]. During γ-radiolysis, the amounts of free radicals are high enough when the competition between oxidation and scavenging determines the method of degradation followed. The differences in the stabilization degrees of polymer composites, including oxides, are ascertained by the filler chemistry based on electronic interactions and the concentration of lattice gaps [57]. The transformation of epoxy resin in composite probes is related to the activity of the filler composition, whose electronic density determines the oxidation level in the aging system due to the unpaired electron of each free radical [58].

The accelerated degradation caused by γ-irradiation is an appropriate way through which the stability of materials may be characterized. Starting from the pristine polymers, like the studied cured epoxy resin samples, their composites may achieve high performances if they contain certain configurations able to subtract reactive radicals from the stage of oxidation [59]. Based on the changes induced by high-energy radiation in epoxy resins [60], the radiolysed composites may gain improved properties because the newly generated structures [61] are able to inhibit their latent reactivity through further deactivation on the surface of filler particles [16,62]. The design and synthesis of oxide-based nanocomposites start from some fundamental backgrounds, which are summarized as follows:

- The degradation starts in the polymer phase, but the spreading of free radicals is hindered by the interaction with the inorganic phase;
- The diffusion of oxygen into the inner part of material is delayed by the oxide particles, which scavenge the free radicals;
- The reorganization of molecular structures must be achieved through a certain degree of crosslinking [63], which promotes high performances in the stability of the product.

These aspects must be taken into consideration as the recycling of waste polymers is a real source of information for several technical articles [64], especially for shielding applications [65].

**Figure 6:**
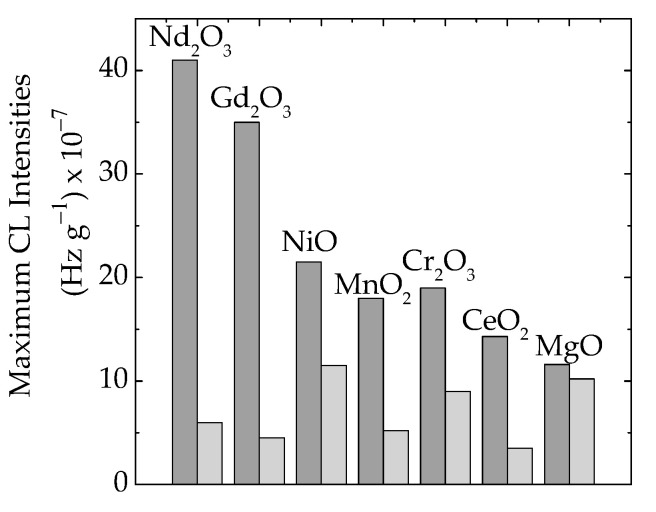
The histogram of maximum emission intensities measured on EPDM/oxide composites in various irradiation states: (dark grey) 0 kGy, (light grey) 50 kGy.

The presence of oxide particles in the formulations of polymer composites is a main factor that influences the material’s functional characteristics [66]. Because the initiation of the thermal oxidation of polymer/oxide composites is influenced by the abundance of free radicals (Figure 2), the degree of curing may delay the propagation of degradation and the material remains less oxidized at temperatures around 70–80 °C. The crosslinking of the EPDM support attains a high level at 100 kGy [67], and a preliminary radiation treatment brings about an advanced stability when hazardous operation conditions are considered. The availability of free radicals born in a polymer matrix is related to the contribution of each type of particle (Figure 2a). This becomes the primary reason, based on the interphase interaction for a large stability range from 55 °C in EPDM/Nd_2_O_3_ to 93 °C in EPDM/MgO. This effect may be determined from the examination of maximum CL intensities (Figure 6), when the scavenging activities and polymer consistencies are correlated with the definition of material stabilities resulting from the consequences of irradiation on the delaying action of fillers.

The analysis on the share of oxide filler in γ-irradiated epoxy resins must be based on the fate of free radicals that appear after molecular breaking and the propagation of the degradation chain through the inter-radical reactions. The differences between the concentrations of intermediates can be determined through the comparison of CL intensities for maximum emissions (Figure 6). These results support the hypothesis that the partial decay of radicals occurs due to recombination and crosslinking instead of oxidation.

The high-energy irradiation of epoxy resin systems highlights the improvement in the mechanical properties [68], thermal performances [54], optical characteristics [69], chemical constitution [37], or structural changes due to molecular fragmentation during γ-radiolysis [70]. All these modifications involve either the retention of radical on the surface of oxide particles or the structuration of further correlation of intermediates into larger molecular structures [71].

**Figure 7:**
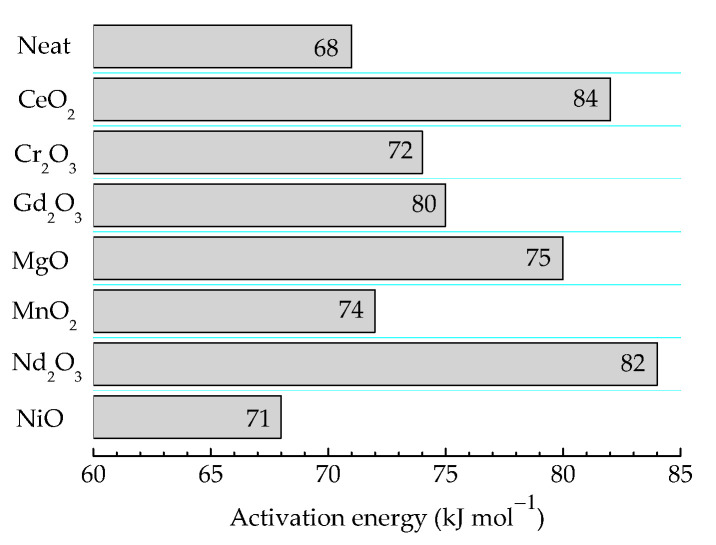
The values of activation energies required for the oxidation of epoxy resin/oxide systems γ-irradiated at 100 kGy.

The durability of products based on γ-irradiated epoxy resins is determined according to the received doses and dose rates. The high values of these technological parameters are essential factors that characterize the oxidation strength and the integrity of the items [72]. The consequences of irradiation processing are tightly related to the implication of filler particles that are able to withdraw free radicals from the degradation chain. Their further state is described by the depth of the interaction, which is measured according to the values of the activation energy required for their oxidation. In Figure 7, these values are illustrative for the contribution of oxide fillers for the protection of polymer substrate against oxidation due to the accelerated aging at 100 kGy. It may normally be supposed that the relative ratios between the presented figures are maintained at higher doses, but the values would be influenced by the radiolysis degree.

The presented energies are in good agreement with the values previously reported for two thermosetting materials based on an epoxy resin structure [55] whose values are in the range of 71–75 kJ mol^−1^. Another former paper describing the pyrolysis decomposition of epoxy resins offers higher values of activation energies (140 kJ mol^−1^) [33]. This value is high enough in comparison with our results because this reported information concerns the full decomposition of resin up to the elementary components (CO and ĊH_3_) at high temperatures (300–450 °C).

The inclusion of metallic oxide powders in the formulations of the composites prepared with epoxy resins allows for the extension of the operation ranges according to the delayed start of oxidation and the decrease in CL emission intensities after the irradiation of the studied samples (Figure 5).

## 5. Conclusions

This study analyses the improvement effects of some oxide fillers on cured epoxy resin subjected to an accelerated degradation caused by the radiation processing in a γ-field. This estimation is a useful assay for the evaluation of protection efficiency for several metallic surfaces, especially the outer parts of products subjected to a sustained energetic transfer. The non-isothermal chemiluminescence measurements, as an accurate approach to the determination of a polymer material’s stability, reveal the scavenging activity on the particle’s surface, where the jointed free radicals are abstracted from the oxidative degradation chain. The added metallic oxide fillers simulate the role of impurities present in any alloy composition and the revealed effect may be ascribed to the contribution of microelements to the passivation processing. The differences that exist in the values of onset oxidation temperatures and emission intensities highlight the importance of electronic densities characterizing the metallic atoms and the various degrees of contribution to the durability of these composites under an advanced oxidation regime. The γ-irradiation of these composite systems, whose oxidation strength depends on the environmental operation conditions, proves that this treatment is an appropriate procedure for increasing oxidation strength as a pre-treatment through which high technological resistance is achieved. The values of activation energies required for the practically ameliorated protection against oxidative aging are reliable proof for the fabrication of long-term products. The association of metallic oxides with an epoxy resin matrix provides interesting compositions for several applications like anticorrosive protection; cases and commodities for electronic and IT equipment, and a safe handling of food; various sizes of spacers for buildings and furniture; thermal insulations for the pipes transporting fluids; and spare parts for automotive.

## Data Availability

The data presented in this study are available on request from the corresponding authors.
